# Parental Relationship with Twins from Pregnancy to 3 Months: The Relation Among Parenting Stress, Infant Temperament, and Well-Being

**DOI:** 10.3389/fpsyg.2016.01628

**Published:** 2016-10-21

**Authors:** Laura E. Prino, Luca Rollè, Cristina Sechi, Luciana Patteri, Anna Ambrosoli, Angela M. Caldarera, Eva Gerino, Piera Brustia

**Affiliations:** ^1^Department of Psychology, University of TorinoTorino, Italy; ^2^Department of Pedagogy, Psychology, and Philosophy, University of CagliariCagliari, Italy

**Keywords:** twins, mother, father, parenting stress, infant temperament, anxiety, depression, dyadic adjustment

## Abstract

**Objective:** The transition to parenthood, from pregnancy to postpartum period, is a critical process, particularly for couples expecting twins. There is very little literature regarding the links between anxiety, depression, dyadic adjustment, parental stress, and infant temperament spanning from pregnancy to postpartum. This study has two aims: first, to examine whether mothers’ and fathers’ anxiety, depression, and dyadic adjustment, assessed at the sixth month of pregnancy and 3 months postpartum, are associated with infants’ negative affectivity (NA) and parenting stress; second, to examine whether there is any difference between fathers’ and mothers’ levels of parenting stress and perception of the twins’ temperament, as well as to evaluate, separately for mothers and fathers, whether the levels of parenting stress and perception of child temperament differ for each twin.

**Method:** The study participants were 58 parents (29 couples) and their healthy 58 twin babies (51.7% boys, 48.3% girls). Mothers’ ages ranged from 30 to 44 years, (*M_Age_* = 36.3 years, *SD* = 3.2 years), and fathers’ ages ranged from 32 to 52 years, (*M_Age_* = 38.2 years, *SD* = 4.4 years). The parents, during the pregnancy period and 3 months after delivery, filled out the Edinburgh Postnatal Depression Scale, the State-Trait Anxiety Inventory, and the Dyadic Adjustment Scale. Three months after delivery they also filled out the Parenting Stress Index—Short Form and the Infant Behavior Questionnaire Revised.

**Results:** The analyses showed a significant correlation between parental anxiety/depression symptoms and infants’ NA and parenting stress (in both mothers and fathers). Moreover, compared to fathers, mothers reported higher scores on specific dimensions of the infants’ NA, [*t*(28) = -2.62 and *p* < 0.05; *t*(28) = 2.09 and *p* < 0.05], and parenting stress, [*t*(28) = 2.19 and *p* < 0.05; *t*(28) = 2.23 and *p* < 0.05], but only for Twin 2. Finally, the results showed that mothers’ perceptions of child temperament vary between two twins, [e.g., distress to limitations: *t*(28) = 2.08 and *p* < 0.05].

**Discussion:** This study highlights the peculiarity of twin parenthood during the fourth trimester. In particular, the differences between twins’ mothers’ and fathers’ perceptions are relevant from a clinical perspective and for perinatal professionals. It would be interesting to study the long-term impact of mothers’ and fathers’ differing perceptions of their twins.

## Introduction

The importance of the transition to parenthood, a “critical” step in most lives, is particularly clear in the case of multiple births (Glazebrook et al., 2004). Multiple birth is a rapidly growing phenomenon; in Italy, the 8,719 multiple births that occurred in 2013 accounted for 1.7% of the total births that year (Basili et al., 2015). Twins pregnancy, as well as twins delivery, falls into the category of “risk pregnancies” because of the high risk of complications during both pregnancy and delivery for the mother and the babies (Brustia et al., 2014). These pregnancies may result in physical and mental complications for both parents and babies (Crosignani and Rubin, 2000; Elster, 2000; Brustia et al., 2008; Taubman-Ben-Ari et al., 2008; Garbarini, 2011; Brustia et al., 2014). Fathers play a very important role in multiple birth because they represent a source of support for the mothers during this peculiar experience (Brustia et al., 2009). The impact of the new birth varies according to several characteristics, such as psychological traits of parents and/or children and the environment in which the new parents and baby are settled (Bornstein and Venuti, 2013).

Despite being characterized as joyful events, twin pregnancy and birth can cause significant vulnerability in future parents (Condon et al., 2004; Glazebrook et al., 2004; Edhborg et al., 2005; Mazzeschi et al., 2015; Anding et al., 2016) and can impact their psychological lives in the form of depression (Matthey et al., 2000; Beeghly et al., 2002; Goodman, 2004; Edhborg et al., 2005; Schumacher et al., 2008; Fisher et al., 2012; O’Hara and McCabe, 2013; O’Hara and Wisner, 2014; Epifanio et al., 2015), anxiety (Matthey et al., 2000; Skari et al., 2002; Buist et al., 2003; Condon et al., 2004; Heron et al., 2004; Edhborg et al., 2005; Andersson et al., 2006; Boyce et al., 2007; Figueiredo and Conde, 2011), parenting stress (Cornish et al., 2006; Leigh and Milgrom, 2008; Misri et al., 2010; Bornstein and Venuti, 2013), or in the couple’s adjustment (Belsky, 1985; Belsky and Isabella, 1985; Belsky et al., 1985; Belsky and Rovine, 1990; Cobb et al., 2008; Lawrence et al., 2008; Garbarini, 2011; Velotti et al., 2011).

Becoming a parent is a period of great vulnerability to psychological disorders (Edhborg et al., 2005; Epifanio et al., 2015). Postpartum depression (PPD) is a perinatal mood disorder (American Psychiatric Association, 2013). A relevant issue concerning PPD is its onset. According to the DSM-IV TR (American Psychiatric Association, 2000), PPD arises within 4 weeks after delivery. Contrastingly, according to the current version of the Diagnostic and Statistical Manual of Mental Disorders (American Psychiatric Association, 2013), PPD might begin either during pregnancy or postpartum. Another difference is that the lapse of time after birth after which PPD might start has been extended from the former 4 weeks after delivery to 6 months after delivery (American Psychiatric Association, 2013). The definition of the postpartum period remains unclear: O’Hara and McCabe (2013) suggest that it can vary according to the purposes of the research. Time frames used to define the postpartum period in clinical practice and in the field of research can vary up to 12 months (O’Hara and McCabe, 2013). PPD can interfere with child development and family wellbeing (O’Hara and Wisner, 2014); it affects approximately 15–20% of mothers (Fisher et al., 2012; O’Hara and McCabe, 2013).

Although PPD research tends to be more focused on the maternal figure, there is a growing awareness that fathers can also experience this mood disorder (Condon et al., 2004; Wee et al., 2011; Epifanio et al., 2015). The incidence of paternal PPD ranges from 1.2% to 25.5% in developed countries (Goodman, 2004). Paternal PPD differs from maternal PPD in several ways (Goodman, 2004; Schumacher et al., 2008). Current literature shows that PPD begins later in fathers than in mothers; it often follows the onset of PPD in the partner (Matthey et al., 2000). PPD in fathers involves high levels of anger, affective rigidity, substance abuse, and other general somatic symptoms (Schumacher et al., 2008; Epifanio et al., 2015). A personal history of depression and the quality of the dyadic relationship are important PPD risk factors, but the strongest one is the simultaneous existence of depression in the partner (Matthey et al., 2000; Goodman, 2004; Epifanio et al., 2015). Indeed, some studies have suggested that PPD in one partner is strongly correlated with PPD in the other partner (Matthey et al., 2000; Dudley et al., 2001; Goodman, 2004; Paulson and Bazemore, 2010; Anding et al., 2016); more specifically, when a mother is severely depressed, the possibility that the father also suffers from PPD doubles (Harvey and McGrath, 1988).

High levels of anxiety represent another disease typical of the perinatal period. Severe anxiety is more widespread than PPD both during pregnancy and after birth in mothers-to-be/new-mothers (Wenzel et al., 2005; Lee et al., 2007). Generally, both women and men experience more severe anxiety during pregnancy than during the postpartum period (Buist et al., 2003; Condon et al., 2004; Heron et al., 2004; Andersson et al., 2006). Current literature points out the frequency of comorbidity of severe anxiety and PPD in the perinatal period. Some studies have suggested that high-level anxiety and depression occur more consistently in women than in men (Matthey et al., 2000; Skari et al., 2002; Edhborg et al., 2005), but we need to consider the fact that only in the last few decades were men included in this family research (Field et al., 2006). In both women and men anxiety levels are higher during early pregnancy than at 3 months postpartum (Andersson et al., 2006; Boyce et al., 2007; Figueiredo and Conde, 2011). In mothers, high levels of anxiety usually decrease from pregnancy to postpartum (Figueiredo and Conde, 2011). To our knowledge, comparable statistically significant data regarding fathers does not exist.

Dyadic adjustment (Spanier, 1979) – a construct composed of consensus on matters of importance to dyadic functioning, dyadic cohesion, dyadic satisfaction, troublesome dyadic differences, and interpersonal tensions and personal anxiety – is another important issue related to parenthood. After birth, relationship satisfaction declines, starting during the last trimester of pregnancy and continuing until the postpartum period (Lawrence et al., 2008). The decline in relationship satisfaction could be due to the changes that occur during the transition to parenthood (e.g., Darwiche et al., 2015), for example, the decrease in time spent together; the decrease in affectional feelings; the decrease in time given to communication and discussion; interference with sexual habits and therefore with intimacy; and the division of roles between housework and child care (Belsky, 1985; Belsky and Isabella, 1985; Belsky et al., 1985; Belsky and Rovine, 1990; Cobb et al., 2008; Velotti et al., 2011). Garbarini et al. (2014) have adapted the Dyadic Adjustment Scale (Spanier, 1979) to the Italian context. The results of the study on Italian couples are consistent with the results of Spanier’s; the amount of marital conflict is the only difference between the two samples. Garbarini (2011) has conducted research regarding dyadic adjustment in Italian non-parents, parents of single-born children, and parents of twins. The findings show that twins’ parents obtained lower total scores on the DAS (Spanier, 1979) than non-parents and parents of single-born children, although this data cannot be considered statistically significant; the non-parents group obtained higher total scores than the other groups. In others words, single-borns’ and twins’ parents’ relationship satisfaction levels, while similar to each other, differed from the non-parents’ levels (Garbarini, 2011).

It is possible to consider both ante- and postpartum relationship satisfaction, as indicated by the score on the DAS, a strong predictor of parenting stress (Ganiban et al., 2007; Mazzeschi et al., 2015). Morse et al. (2000) suggest that a poor relationship mid-pregnancy can also predict higher vulnerability to postnatal stress. A low score on the DAS after birth is often associated with less functional parenting than that of a high-scoring couple (Krishnakumar and Buehler, 2000; Ganiban et al., 2007). Conversely, the proper level of relationship satisfaction can function like a “buffer” that can prevent the onset of parenting stress (Goldberg and Smith, 2014).

Parenting stress can be defined as the discrepancy between the required resources of the parental role and the perceived resources available to meet those requests (Abidin, 1995). Parenting stress is the result of characteristics of both parent and child and their interaction (Misri et al., 2010). Some studies have shown that depression and high levels of anxiety in the third trimester of pregnancy can predict the onset of parenting stress in mothers at either 3 or 6 months postpartum (Cornish et al., 2006; Leigh and Milgrom, 2008; Misri et al., 2010). Parental stress (Leigh and Milgrom, 2008; Misri et al., 2010; Mazzeschi et al., 2015) can be predicted by maternal depression and anxiety during pregnancy, although postnatal depression is the stronger predictor (Mazzeschi et al., 2015). To our knowledge, there are few studies that explore the effects of paternal PPD (Misri et al., 2010), and no statistically significant data which can prove the correlation between paternal PPD and parenting stress exists.

Both parents’ and children’s characteristics can be considered risk factors for parenting stress (Bornstein and Venuti, 2013; Scarzello and Prino, 2015). Child temperament plays a crucial role in parenting. It is possible to define temperament as the individual characteristics of the child, determined by regularity in attitude, eating, sleeping, adapting to changes in his/her environment, and eliminating reaction to stimuli (Bates et al., 1979). The issue when dealing with child temperament is the validity of caregivers’ reports (Bates, 1980), specifically of parents’ agreeing reports of child temperament. Most studies featuring a high level of agreement have not eliminated the possibility of an earlier discussion between parents about the behavior of the baby (Bates, 1980). To our knowledge, there are no studies that show differences between mothers’ and fathers’ perceptions of child temperament. Even in this study, the results showed no differences between maternal and paternal perception. With regard to multiple births, the literature indicates that there are some differences between parents’ perceptions of monozygotic and dizygotic twins’ temperaments (Saudino et al., 2000). According to Saudino et al. (2000), parents tend to describe monozygotic twins as identical in behavior, whereas dizygotic twins are described as completely different.

Based on the above empirical and clinical evidence, the present longitudinal study had the following aims:

1 To examine whether mothers’ and fathers’ anxiety, depression, and dyadic adjustment, assessed at the sixth month of pregnancy and at 3 months postpartum (Times 1 and 2, respectively), are associated with the infants’ negative affectivity (NA) and with parenting stress (Time 2).
2 To examine whether there are any differences between fathers’ and mothers’ levels of parenting stress and perception of the temperament of the twins, as well as to test, separately for mothers and fathers, if the levels of parenting stress and perception of child temperament are different for each twin.

This study was part of a larger, ongoing longitudinal study on maternal and paternal depression in first-time parents and the development of their children’s affective regulation. In this paper, we present data concerning parents who completed the first (Time 1) and second step (Time 2) of the assessment during the pregnancy and third month after the child’s birth.

## Materials and Methods

### Participants

The study participants were 58 primiparous parents (29 couples) and their healthy 58 twin babies (51.7% boys, 48.3% girls). Of these, 100% were married couples; 10% of the fathers had an elementary school qualification, 24% of the mothers and 35% of the fathers had a high-school qualification, 62% of the mothers and 48% of the fathers had a college degree, and 14% of the mothers and 7% of the fathers had a PhD. Mothers’ ages ranged from 30 to 44 years (*M_Age_* = 36.3 years, *SD* = 3.2 years), and fathers’ ages ranged from 32 to 52 years (*M_Age_* = 38.2 years, *SD* = 4.4 years). The median income of the parents belonged to the Italian middle working class and socio-economic status as assessed by a detailed questionnaire and according to ISTAT classification (Istituto Nazionale di Statistica [ISTAT], 2013). No participant was undergoing medical/psychological treatment at the time of assessment.

### Measures

The *Edinburgh Postnatal Depression Scale* (EPDS; Cox et al., 1987) is a self-report questionnaire including ten items that address depression symptoms occurring within the previous 7 days. The total score is calculated by adding the individual items on a four-point Likert-style scale. There were two adopted cut-off scores: 8/9, as suggested in the EPDS Italian validation (Benvenuti et al., 1999), and 12/13, as suggested by Cox et al. (1987), to identify more severe depression. In the current study, the internal consistency coefficient for the mothers was α = 0.82 during pregnancy and α = 0.84 at 3 months; for the fathers, it was α = 0.78 during pregnancy and α = 0.74 at 3 months.

The *State-Trait Anxiety Inventory* (STAI; Spielberger et al., 1983; Pedrabissi and Santinello, 1989) is a commonly used self-report measure of trait and state anxiety. STAI has 20 items for assessing trait anxiety (STAI-T) and twenty for state anxiety (STAI-S). All items are rated on a four-point scale (i.e., from “Almost Never” to “Almost Always”). The adopted cut-off score was >40, as suggested by the Italian validated version (Pedrabissi and Santinello, 1989). In the current study, the internal consistency coefficient for the STAI-S was α = 0.93 during pregnancy and α = 0.89 at 3 months for the mothers; for the fathers, it was α = 0.94 during pregnancy and α = 0.87 at 3 months. The internal consistency coefficient for the STAI-T was α = 0.87 during pregnancy and α = 0.86 at 3 months for the mothers; for the fathers, it was α = 0.94 during pregnancy and α = 0.94 at 3 months.

The *Dyadic Adjustment Scale* (DAS; Spanier, 1976; Gentili et al., 2002; Garbarini et al., 2014) is a 32 items for assessing dyadic adjustment in couples. The total score is a composite of the subscale scores: dyadic consensus, affectional expression, dyadic satisfaction, and dyadic cohesion.

The levels of dyadic adjustment were assessed using the cut-off scores reported by Graham et al. (2006); scores below 100 are considered to indicate distress, while scores above 107 indicate satisfaction.

In this research, the internal consistency coefficient for the mothers was α = 0.83 during pregnancy and α = 0.86 at 3 months; for the fathers, it was α = 0.84 during pregnancy and α = 0.87 at 3 months.

The *Parenting Stress Index—Short Form* (PSI-SF; Abidin, 1995; Guarino et al., 2008) is a self-report instrument that measures stress specifically associated with parenting. The PSI-SF consists of 36 statements referring to the past week. All items are rated on a five-point scale. Parents who obtain a total stress score above the 90th percentile or a raw score of 90 are considered to experience clinically significant parenting stress, as indicated by the Italian validation (Guarino et al., 2008). The total stress score is a composite score of the subscale scores: parental distress, parent–child dysfunctional interaction, and difficult child. In the current study, the internal consistency coefficient for the mothers was α = 0.92 and α = 0.91 for Twins 1 and 2, respectively; for the fathers, it was α = 0.95 and α = 0.95 for Twins 1 and 2 respectively.

The *Infant Behavior Questionnaire* (IBQ-R; Gartstein and Rothbart, 2003; Montirosso et al., 2010) is a 191-item parent-report measure of temperament designed for use with infants between ages 3 and 12 months. The IBQ-R yields fourteen subscales subsumed by three higher-order factors: Surgency/Extraversion (SE; approach, vocal reactivity, high intensity pleasure, smiling and laughter, activity level, and perceptual sensitivity), NA (sadness, distress to limitation, fear, and low-falling reactivity), and Orienting/Regulatory Capacity (ORC; low intensity pleasure, cuddliness, duration of orienting, and soothability).

Mothers and fathers rated the frequency of infant behaviors on a scale from a 1 (never) to 7 (always). In the current study, internal consistency coefficient was calculated for the three overarching factor scores of the IBQ-R for both mothers and fathers.

The alpha coefficient(s) for SE was α = 0.97 (Twin 1) and α = 0.98 (Twin 2) for the mothers; for the fathers, it was α = 0.84 (Twin 1) and α = 0.85 (Twin 2).

The alpha coefficient(s) for NA was α = 0.97 (Twin 1) and α = 0.98 (Twin 2), for mothers; for the fathers, it was α = 0.79 (Twin 1) and α = 0.87 (Twin 2).

The alpha coefficient(s) for ORC was α = 0.96 (Twin 1) and α = 0.97 (Twin 2), for mothers; for the fathers, it was α = 0.94 (Twin 1) and α = 0.87 (Twin 2).

### Procedure

The research project obtained approval from the University of Torino bioethics committee. All participants signed a written informed consent form and received an informative sheet on the research.

Between the 24–26th weeks of pregnancy (Time 1), mothers and fathers independently filled out: the State-Trait Anxiety Inventory (STAI; Spielberger et al., 1983), the EPDS (Cox et al., 1987), and the Dyadic Adjustment Scale (DAS; Spanier, 1976). Three months after the children’s birth (Time 2), mothers and fathers were again asked to fill out the EPDS, the STAI, and the DAS, and completed the Parenting Stress Index (PSI; Abidin, 1995) and the Infant Behavior Questionnaire (IBQ-R; Gartstein and Rothbart, 2003).

The parents were informed that the mother could decide which child’s individual IBQ and PSI would be filled out first; in this case, the child labeled “first child” for the mother would have been the “first child” also for the father.

### Data Analysis

Data analysis was conducted with IBM SPSS Version 21.

The data was preliminarily screened for errors and outliers. No missing data was detected.

Descriptive statistics were calculated on the assessed psychological variables, reporting frequencies, percentages, mean values, and standard deviations. Before performing the analyses, the Shapiro–Wilks test for normality was performed; all the variables were normally distributed.

Pearson’s correlations were used to assess the association between mothers’ or fathers’ anxiety/depression symptoms, marital distress, infants’ NA, and parenting stress.

Since the mother and the father in a couple were considered to be dependent, all comparisons between mothers and fathers used statistical methods for paired data. To analyze the differences between fathers’ and mothers’ scores of parenting stress and perceptions of infants’ temperaments, we used a paired sample *t*-test. Finally, to analyze the levels of parenting stress produced by Twins 1 and 2 and the perception of temperaments of Twins 1 and 2 on each parent separately, we used a paired sample *t*-test.

## Results

### Descriptive Analyses

Means and standard deviations of each variable used in this study were calculated and the percentages of mothers and fathers experiencing clinically significant depression, anxiety, marital distress, and parenting stress levels are reported in **Table 1**.

**Table 1:** Frequency distribution of EPDS, STAI, DAS and PSI for mothers and fathers.

	Time 1 (Pregnancy)		Time 2 (3 Months)	
	Mothers	Fathers	Mothers	Fathers
**EPDS**				
Borderline (9–1*2)* N (%)	3 (10.3%)	–	6 (20.7%)	2 (6.9%)
Depressed (≥13) N (%)	–	1 (3.4%)	3 (10.3%)	–
**STAI**				
**STAI – State**				
Anxious (>40) N (%)	7 (24.1%)	2 (6.9%)	5 (17.2%)	4 (13.8%)
**STAI – Trait**				
Anxious (>40) N (%)	6 (20.7%)	1 (3.4%)	5 (17.2%)	4 (13.8%)
**DAS**				
**Total score**				
Distress (<100) N (%)	2 (6.9%)	2 (6.9%)	3 (10.3%)	3 (10.3%)
**PSI**				
**Total stress twin 1**				
Clinical stress (>90) N (%)	–	–	1 (3.4%)	2 (6.9%)
**Total stress twin 2**				
Clinical stress (>90) N (%)	–	–	2 (6.9)	2 (6.9)

*EPDS, Edinburgh Postnatal Depression Scale; STAI, State and Trait Anxiety Inventory; DAS, Dyadic Adjustment Scale; PSI, Parenting Stress Index-Short Form.*

### Association between Mothers’ and Fathers’ Anxiety, Depression, Marital Distress, Infants’ Negative Affectivity, and Parenting Stress

**Tables 2** and **3** (respectively) show the bivariate correlations between mothers’ and fathers’ anxiety/depression symptoms, marital distress, infants’ NA, and parenting stress.

**Table 2:** Bivariate correlations among study variables for mothers.

Mothers	EPDS T1	EPDS T2	STAI S T1	STAI S T2	STAI T T1	STAI T T2	DAS T1	DAS T2
P-STRESS *TW1*	0.10	0.61**	0.39*	0.68**	0.29	0.70**	-0.12	-0.30
P-STRESS *TW2*	-0.04	0.54**	0.21	0.62**	0.36	0.61**	-0.23	-0.36
IBQ-R *Distress TW1*	0.02	0.50**	0.26	0.54**	0.36	0.57**	-0.37	-0.22
IBQ-R *Distress TW2*	-0.06	0.58**	0.24	0.61**	0.35	0.53**	-0.34	-0.30
IBQ- R *Fear TW1*	-0.02	0.24	0.22	0.08	0.24	0.07	-0.16	-0.02
IBQ- R *Fear TW2*	-0.18	0.20	0.02	0.05	0.20	0.06	0.01	-0.06
IBQ-R *Falling reactivity TW1*	-0.04	-0.27	0.09	-0.33	-0.33	-0.32	0.12	0.11
IBQ-R *Falling reactivity TW2*	0.16	0.30	0.06	-0.30	-0.23	-0.25	0.21	0.30
IBQ-R *Sadness TW1*	0.20	0.43*	0.29	0.47*	0.40*	0.58**	-0.22	-0.05
IBQ-R *Sadness TW2*	0.01	0.58**	0.35	0.72**	0.56**	0.68**	-0.32	-0.29

*p < 0.05, **p < .01. EPDS T1, Edinburgh Postnatal Depression scores during pregnancy; EPDS T2, Edinburgh Postnatal Depression scores at three months after the children’s birth; STAI S T1, State Anxiety Inventory scores during pregnancy; STAI S T2, State Anxiety Inventory scores at three months after the children’s birth; STAI T T1, Trait Anxiety Inventory scores during pregnancy; STAI T T2, Trait Anxiety Inventory scores at three months after the children’s birth; DAS T1, Dyadic Adjustment scores during pregnancy; DAS T2, Dyadic Adjustment scores at three months after the children’s birth; P-STRESS TW1, Parenting Stress Index Total Stress scores for Twin 1; P-STRESS TW2, Parenting Stress Index Total Stress scores for Twin 2; IBQ-R Distress TW1, Infant Behavior Questionnaire-Revised: distress to limitations scores of Twin 1; IBQ-R *Distress TW2*, Infant Behavior Questionnaire-Revised: distress to limitations scores of Twin 2; IBQ- R *Fear TW1*, Infant Behavior Questionnaire-Revised: Fear scores of Twin 1; IBQ- R *Fear TW2*, Infant Behavior Questionnaire-Revised: Fear scores of Twin 2; IBQ- R *Falling reactivity TW1*, Infant Behavior Questionnaire-Revised: Falling reactivity scores of Twin 2; IBQ- R *Falling reactivity TW2*, Infant Behavior Questionnaire-Revised: Falling reactivity scores of Twin 2; IBQ- R *Sadness TW1*, Infant Behavior Questionnaire-Revised: R Sadness scores of Twin 1; IBQ- R *Sadness TW2*, Infant Behavior Questionnaire-Revised: R Sadness scores of Twin 2.

**Table 3:** Bivariate correlations among study variables for fathers.

Fathers	EPDS T1	EPDS T2	STAI S T1	STAI S T2	STAI T T1	STAI T T2	DAS T1	DAS T2
P-STRESS *TW1*	0.59**	0.69**	0.43*	0.74**	0.43*	0.81**	-0.33	-0.65**
P-STRESS *TW2*	0.58**	0.61**	0.44*	0.83**	0.44*	0.83**	-0.35	-0.64**
IBQ-R *Distress TW1*	0.06	0.10	-0.16	0.20	-0.14	0.27	0.16	0.07
IBQ-R *Distress TW2*	0.30	0.43*	0.50**	0.66**	0.42*	0.49**	-0.37	-0.32
IBQ- R *Fear TW1*	-0.15	-0.20	-0.11	-0.09	-0.17	-0.06	0.30	0.35
IBQ- R *Fear TW2*	0.10	0.16	0.25	0.25	0.16	0.07	-0.32	-0.12
IBQ-R *Falling reactivity TW1*	-0.05	-0.14	0.06	-0.14	0.03	-0.22	0.06	-0.01
IBQ-R *Falling reactivity TW2*	-0.42*	-0.49**	-0.42*	-0.59**	-0.36	-0.49**	0.22	0.21
IBQ-R *Sadness TW1*	-0.21	-0.02	-0.25	-0.11	-0.15	0.05	0.20	0.07
IBQ-R *Sadness TW2*	0.39*	0.64**	0.68**	0.64**	0.54**	0.57**	-0.32	-0.36

*p < 0.05, **p < 0.01.

*EPDS T1, Edinburgh Postnatal Depression scores during pregnancy; EPDS T2, Edinburgh Postnatal Depression scores at three months after the children’s birth; STAI S T1, State Anxiety Inventory scores during pregnancy; STAI S T2, State Anxiety Inventory scores at three months after the children’s birth; STAI T T1, Trait Anxiety Inventory scores during pregnancy; STAI T T2, Trait Anxiety Inventory scores at three months after the children’s birth; DAS T1, Dyadic Adjustment scores during pregnancy; DAS T2, Dyadic Adjustment scores at three months after the children’s birth; P-STRESS TW1, Parenting Stress Index Total Stress scores for Twin 1; P-STRESS TW2, Parenting Stress Index Total Stress scores for Twin 2; IBQ-R *Distress TW1*, Infant Behavior Questionnaire-Revised: distress to limitations scores of Twin 1; IBQ-R *Distress TW2*, Infant Behavior Questionnaire-Revised: distress to limitations scores of Twin 2; IBQ- R *Fear TW1*, Infant Behavior Questionnaire-Revised: Fear scores of Twin 1; IBQ- R *Fear TW2*, Infant Behavior Questionnaire-Revised: Fear scores of Twin 2; IBQ- R *Falling reactivity TW1*, Infant Behavior Questionnaire-Revised: Falling reactivity scores of Twin 2; IBQ- R *Falling reactivity TW2*, Infant Behavior Questionnaire-Revised: Falling reactivity scores of Twin 2; IBQ- R *Sadness TW1*, Infant Behavior Questionnaire-Revised: R Sadness scores of Twin 1; IBQ- R *Sadness TW2*, Infant Behavior Questionnaire-Revised: R Sadness scores of Twin 2.*

A significant correlation emerged (**Table 2**) between mothers’ anxiety trait during pregnancy, anxiety/depression at 3 months postpartum, and specific dimensions of the infants’ NA (distress to limitations and sadness). These correlations were higher for Twin 2 than for Twin 1. Significant correlations were also found between higher anxiety/depression scores at 3 months postpartum and parenting stress. Infants’ NA and parenting stress scores were not significantly associated with marital distress.

For fathers (**Table 3**), at the bivariate level, higher scores in anxiety/depression during pregnancy and at 3 months postpartum correlated significantly to only specific dimensions of Twin 2’s infant NA (distress to limitations, low falling reactivity, and sadness). Also, a significant correlation emerged between higher anxiety/depression scores during pregnancy and 3 months postpartum and parenting stress. Finally, lower DAS scores at 3 months postpartum correlated significantly with higher parenting stress.

### Comparisons between Fathers’ and Mothers’ Levels of Parenting Stress and Perceptions of Infants’ Temperaments

Differences between the parental distress subscale and total stress score of each partner’s PSI report were found for Twin 2; compared to their partners, mothers reported higher levels of psychological distress and total stress.

The IBQ-R results found differences between each partner’s rating of Twin 2’s infant NA; compared to their partners, mothers perceived their Twin 2 as more distressed to limitations and with a smaller rate of recovery from peak distress (**Table 4**).

**Table 4:** Distribution of outcomes by mother and father couples.

	Twin 1			Twin 2		
	Mothers	Fathers	*t*	Mothers	Fathers	*t*
	Mean score *(SD)*	Mean score *(SD)*		Mean score *(SD)*	Mean score *(SD)*	
**PSI**						
PD	23.7 (7.5)	21.4 (7.7)	1.57	22.7 (6.2)	20.2 (7)	2.05
PCDI	16.7 (4.3)	16 (4.4)	0.90	17.9 (4.6)	15.8 (4)	2.19*
DC	20.5 (7.8)	19.4 (6.5)	0.93	21.2 (7.1)	19.9 (7.2)	1.14
Total stress	60.9 (16.5)	56.8 (16.7)	1.5	61.8 (15.2)	55.9 (16.3)	2.23*
**IBQ-R**						
approach	5 (0.8)	4.9 (0.8)	0.52	4.8 (0.8)	4.9 (0.9)	-0.43
Vocal reactivity	4.3 (0.8)	4.4 (1)	-0.08	4.6 (0.9)	4.5 (1)	0.28
High pleasure	5.5 (0.8)	5.4 (1.1)	0.33	5.5 (0.8)	5.5 (1)	-0.44
Smile and laughter	4.4 (0.8)	4.5 (0.9)	-0.60	4.1 (0.89	4.3 (0.9)	-1.13
Activity level	3.5 (0.7)	3.5 (1)	-0.24	3.8 (0.9)	3.8 (1)	0.40
Perceptual sensitivity	3.6 (1.2)	3.9 (81.3)	-0.99	3.2 (1)	3.6 (1.2)	-1.22
Sadness	3.3 (1.1)	3.1 (0.9)	0.67	3.4 (1.1)	3.1 (0.9)	1.61
Distress to limitations	3.4 (0.9)	3.3 (0.1)	0.53	3.7 (1)	3.3 (1.1)	2.09*
Fear	2.2 (0.6)	2.3 (0.8)	-0.84	2.2 (0.8)	2.3 (0.8)	-0.82
Falling reactivity	4.9 (0.9)	5.2 (0.9)	-1.4	4.8 (0.1)	5.2 (0.8)	-2.62*
Low pleasure	5.5 (0.9)	5.5 (0.7)	-0.16	5.1 (0.9)	5.3 (0.9)	-1.23
Cuddliness	6 (0.5)	5.8 (0.7)	1.45	5.9 (0.7)	5.9 (0.7)	-0.09
Duration of orienting	4 (0.8)	4.1 (1)	-0.42	4 (1.2)	4.1 (1.2)	-0.48
Soothability	5.1 (0.7)	5.1 (0.7)	0.12	4.9 (0.7)	5.1 (0.7)	-1.15

*p < 0.05. PSI, Parenting Stress Index—Short Form; PD, Parental Distress; P-CDI, Parent–Child Dysfunctional Interaction; DC, Difficult Child. IBQ-R, Infant Behavior Questionnaire-Revised.

### Comparisons between Twin 1 and Twin 2 for Mothers and Fathers

Because the levels of parenting stress and the perception of the temperaments of each twin differed between the mothers and fathers, we investigated the levels of parenting stress produced on each parent by Twins 1 and 2 and the perceptions of the temperaments of Twins 1 and 2 separately.

A paired sample *t*-test showed differences between mothers’ perceptions of Twins 1 and 2 with respect to activity level (SE) [*t*(28) = -2.22 and *p* < 0.05], distress to limitations (NA) [*t*(28) = -2.20 and *p* < 0.05] and low intensity pleasure (ORC) [*t*(28) = 3.28 and *p* < 0.01]. Compared to Twin 1, the mothers tend to perceive Twin 2 as more distressed to limitations, less able to enjoy to low intensity stimulus, and having higher gross motor activity (**Table 4**).

A paired sample *t*-test showed no differences between fathers’ perceptions of Twin 1 and Twin 2’s temperaments and parenting stress.

## Discussion

Our study furthers the literature on the transition to parenthood in first-time mothers and fathers of twins. The transition to twin parenthood is not very deeply investigated from a psychological point of view in preexisting research, even if it is well-known as a critical moment for the parents. Twinship requires more resources and more involvement of both parents (Brustia et al., 2014). In our study, we considered two periods of time (6 months during pregnancy and 3 months after delivery) and examined anxiety, depression, and relationship satisfaction at both junctures. Parenting stress and infants’ temperaments were assessed in the second step in order to study the association between these variables.

We wish to underscore the importance of distinguishing between motherhood and fatherhood when considering parenthood, as there are different associations between the two situations. For mothers, the results point to a pattern of depression and anxiety at 3 months of parenthood. This seems be to a common pattern in mothers of singletons, as reported by other scientific literature (Matthey et al., 2000; Epifanio et al., 2015; Vismara et al., 2016). For mothers, anxiety during pregnancy is also related to their perception of some infants’ temperamental traits (distress to limitations and sadness), as these were higher for Twin 2. Contrary to what was reported in previous studies (i.e., Mazzeschi et al., 2015), we have found no significant correlation between relationship satisfaction and the other studied dimensions. It would be interesting to consider in more depth if this absence is due to the distribution of the total scores of the DAS, or to the number of participants in our study, or if it is unique to mothers of twins. To date, it is not possible to suggest any hypothesis, because, to our knowledge, there are no studies that consider relationship satisfaction in twins’ parents specifically.

The parenting stress at 3 months after delivery is related only to anxiety and depression measured in the same time period, in accordance with other studies (Cornish et al., 2006; Leigh and Milgrom, 2008; Misri et al., 2010).

The data shows that, for fathers, anxiety and depression, both during their partners’ pregnancy and after delivery, are correlated with parental stress, and that that has, in turn, a negative correlation with the dyadic and marital adjustment (Ganiban et al., 2007; Goldberg and Smith, 2014; Zerach and Magal, 2016). Regarding the temperamental traits of the twins, there is a correlation between some negative affective aspects, (i.e., distress to limitations, low falling reactivity, and sadness), and anxiety and depression pre- and post-delivery, but only for Twin 2.

The mothers’ as well as the fathers’ results on the temperamental traits reports show a particular constellation of Twin 2, (again, the choice to label the twins by number was given to the mothers), that should be further analyzed in future studies.

One concern of our second goal is the comparison of parenting stress and infant temperament of each twin as perceived by both parents.

There are no differences between mothers’ and fathers’ parenting stress for the twin labeled Twin 1 by the mother. However, the mothers reported higher psychological distress and total stress for the twin labeled Twin 2. The same discrepancy between twins is present in perceptions of temperament: the mothers describe Twin 2 with more aspects of NA, including higher distress to limitations and less ability to recover from peak distress.

The perception of the temperaments of the twins reported by the mothers shows some significant differences between Twins 1 and 2; the mothers tend to perceive their Twin 2 as more distressed to limitations, with less amount of enjoyment related to low intensity stimulus characteristics, and with a higher gross motor activity. There are no differences in fathers’ perceptions of the temperaments of the two children.

It seems that mothers and fathers view Twin 1 similarly and view Twin 2 differently. This discrepancy is directly related to the level of parenting stress that the mother experiences from Twin 2. The fathers describe the children as “quite identical” and they have similar stress levels from each twin. This result seems in line with the representation of twinship that views the two children as a unit with no distinction between the two (Zazzo, 1985). It would be interesting in future studies to analyze if there are differences in the cases of monozygotic and dizygotic twins, investigating whether or not body similarity is a factor impacting the fathers’ perception.

Mothers seem to be more able to recognize the individuality of each child as early as 3 months after delivery. This could be the result of the fact that, on average, they spend more time caring for and nurturing the infant than fathers do, even if in the last few decades fathers have lost the role of the breadwinner and have become more and more involved in the everyday lives of their children (Hall, 2005; Craig, 2006; Lamb, 2010; Bertone et al., 2015; Scarzello et al., 2016).

Our results highlight an important issue for future research because they are also related to the distress perceived by mothers. Many studies underline how parental stress and infant temperament can impact the relationship between the parent and the child, the developmental trajectory, and the couple’s satisfaction (Saudino et al., 2000; Neece et al., 2012; Bornstein and Venuti, 2013; Mazzeschi et al., 2015; Scarzello and Prino, 2015). The findings of this study make clear the importance of continuing with such longitudinal research, adding observational data about mothers’ sensitivity and the quality of couples’ relationships in order to evaluate whether these differences are continuously present and how they are related to the personal and relational development.

One limitation of this study is the small number of participants that have been involved in the research that it is strongly related to the incidents of twin’s pregnancy (1.7% of the total birth). Secondly, the self-report measures utilized could have been biased. The fact that the parents agreed voluntarily to be involved in the research might have introduced bias as well.

## Conclusion

We believe that our results are important from a clinical perspective, in terms of increasing the awareness of the clinician and perinatal staff about the peculiarity of twinship and of twin parenthood. Following these results, it would be interesting to study the developmental impact of this difference in mothers’ and fathers’ perceptions of their twins.

## Author Contributions

LP prepared the study design, organized the sample recruitment, collected data, and contributed to the writing of the manuscript’s introduction, discussion, and references sections. LR prepared the study design, organized the sample recruitment, collected data, and contributed to the writing of the manuscript’s introduction, discussion, and references sections. CS prepared the data set, performed statistical analysis, prepared the tables, and contributed to the writing of the methods and results sections. LP organized sample recruitment, collected data, and contributed to the writing of the manuscript’s introduction and references sections. AA prepared the study design, organized the sample recruitment, and collected data. AC organized the sample recruitment and collected data. EG organized the sample recruitment and collected data. PB prepared the study design and supervised the research team.

## Conflict of Interest Statement

The authors declare that the research was conducted in the absence of any commercial or financial relationships that could be construed as a potential conflict of interest.
